# A review of granulocyte colony-stimulating factor receptor signaling and regulation with implications for cancer

**DOI:** 10.3389/fonc.2022.932608

**Published:** 2022-08-11

**Authors:** Sungjin David Park, Apryl S. Saunders, Megan A. Reidy, Dawn E. Bender, Shari Clifton, Katherine T. Morris

**Affiliations:** ^1^ Department of Surgery, University of Oklahoma Health Science Center, Oklahoma City, OK, United States; ^2^ Department of Information Management, University of Oklahoma Health Science Center, Oklahoma City, OK, United States

**Keywords:** CSF3R, GCSFR, regulation, cancer, signaling/signaling pathways

## Abstract

Granulocyte colony-stimulating factor receptor (GCSFR) is a critical regulator of granulopoiesis. Studies have shown significant upregulation of GCSFR in a variety of cancers and cell types and have recognized GCSFR as a cytokine receptor capable of influencing both myeloid and non-myeloid immune cells, supporting pro-tumoral actions. This systematic review aims to summarize the available literature examining the mechanisms that control GCSFR signaling, regulation, and surface expression with emphasis on how these mechanisms may be dysregulated in cancer. Experiments with different cancer cell lines from breast cancer, bladder cancer, glioma, and neuroblastoma are used to review the biological function and underlying mechanisms of increased GCSFR expression with emphasis on actions related to tumor proliferation, migration, and metastasis, primarily acting through the JAK/STAT pathway. Evidence is also presented that demonstrates a differential physiological response to aberrant GCSFR signal transduction in different organs. The lifecycle of the receptor is also reviewed to support future work defining how this signaling axis becomes dysregulated in malignancies.

## Introduction

Granulocyte colony-stimulating factor (GCSF) is a pleiotropic cytokine expressed by the gene transcript *CSF3*. GCSF is a hematopoietic growth factor that regulates the viability, proliferation, and differentiation of granulocytic precursors and the function of neutrophils by signaling through its receptor granulocyte colony-stimulating factor receptor (GCSFR) encoded by *CSF3R*. Both GCSF and GCSFR play important roles as chemical mediators that regulate immune cell homeostasis and coordinators of signal-dependent and non-specific immune responses upon microbial invasion. Cytokine signaling contributes to the effective first line of chemical defense against microbial invasion, resulting in chemotactic signaling to recruit neutrophils and natural killer cells to circulate in the blood and extravasate into interstitial spaces and epithelial surfaces. Because GCSF increases neutrophil mobilization and maturation, a recombinant human GCSF (rh-GCSF) has been used in clinical practice to prevent and treat neutropenia. In this capacity, it has proven highly effective in decreasing the frequency of febrile neutropenia among patients undergoing cytotoxic chemotherapy (1, 2). Consequently, the effects of GCSF on granulopoietic mobilization and differentiation have been evaluated extensively. However, investigators have also begun research into potentially unanticipated pro-tumor effects of GCSF in patients with malignancy, given the development of a broader understanding of the effects of this cytokine on non-immune cells. Recent studies have uncovered a potential oncogenic role for aberrant GCSFR expression and signaling in many hematologic malignancies and several solid cancers. Specifically, overexpression of GCSFR has been identified in nasopharyngeal, oral cavity, breast, colorectal, and ovarian cancer cells with data suggesting a potential role for GCSFR in cancer progression (3–6). Furthermore, an emerging body of evidence suggests that tumor microenvironments are regulated by increased GCSF signaling between tumor cells and adjacent immune cells in the development and progression of gastrointestinal (GI) cancers, which have also been noted to have increased GCSFR expression (7, 8).

Due to the evidence suggesting pro-tumor effects from dysregulated GCSFR signaling, here we seek to summarize what is known about GCSFR structure, signaling, and processing to inform future studies of the role played by GCSF in cancer. Studies performed in healthy cells are leveraged to further understand how the signal transduction pathways that GCSFR stimulates in normal tissues are co-opted in cancer cells. Increased understanding of the regulatory effect of GCSFR on cellular proliferation response patterns is important to guide additional studies into GCSFR’s contribution to oncogenesis and progression of malignancies. This review will discuss recent advances in our understanding of the mechanisms behind the receptor-driven signal transduction in various organ systems and cancerous cell lines to further understand the link between the upregulation of GCSFR and cancer pathogenesis.

## Structure

**Figure 1:**
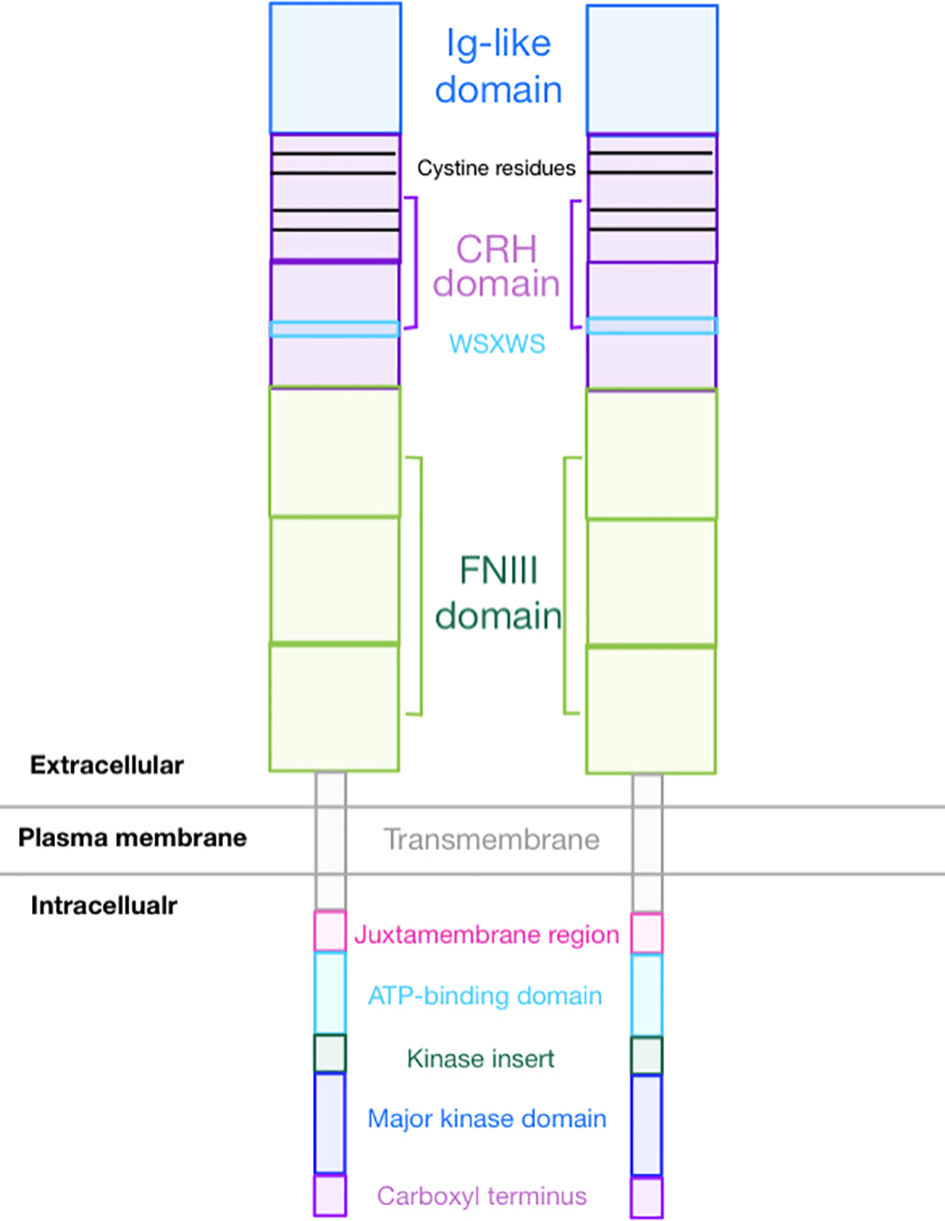
Overall GCSFR Structure.

GCSFR is an 813-amino acid protein encoded by *CSF3R* gene and is a member of the class I cytokine receptor family (9). The receptor is a single transmembrane protein comprised of several functional extracellular and intracellular domains. As seen in **Figure 1**, the extracellular region contains an immunoglobulin (Ig)-like domain, a cytokine receptor homologous (CRH) domain, and three fibronectin type III (FNIII) domains (10). The intracellular region contains three distinct motifs called Box 1, Box 2, and Box 3 and four tyrosine residues (704, 729, 744, and 764) that are essential for mitogenic signal transduction. GCSF requires four highly conserved cysteine residues in the N-terminal half region and the WSXWS motif in the cytokine receptor-homologous (CRH) domain to bind GCSFR and initiate signal transduction (11). Additionally, these four cysteine residues in combination with an additional four cysteine residues at the N-terminal provide eight potential sites for N-linked glycosylation (12).

### Isoforms

Seven messenger RNA (mRNA) isoforms (Class I through VII) can result from alternative splicing of *CSF3R*. While it is unclear which isoforms are expressed in non-hematopoietic cells, the only class I (the canonical type) and class IV (differentiation defective) GCSFR isoforms are detectable in hematopoietic cells (13). Functional mapping studies highlight the importance of the 87-amino acid residues of the carboxy-terminal region in the receptor that allow the signaling for cellular maturation and the 96-amino acid residues of the proximal membrane region that allow the signaling for cellular proliferation. Class IV GCSFR, which is expressed prominently in patients with acute myeloid leukemia (AML), contains a truncation of 87 amino acids at position 725 of the C-terminal along with dileucine residues required for normal receptor internalization, which are replaced by a unique 34-amino acid sequence (9, 14). These changes are thought to result in receptor overexpression due to a lack of normal internalization. Increased expression of Class IV GCSFR has also been linked to increased incidence of AML relapse (15).

## Signal transduction

The immune response to pathogenic microbial invasions is triggered by the finely tuned cascading signal transductions of neighboring cells. GCSF and GCSFR play an integral role in an adaptive immune response through their immunomodulation effect. However, many investigators have also found that GCSFR signaling is increased in multiple cancers as compared to expression levels in healthy cells.

Activation of the receptor enhances the rate of cellular proliferation through the initiation of a cascade of intracellular signaling that is propagated by many factors, among which are Src and a tyrosine kinase protein, Janus Kinase (JAK). This results in the downstream activation of the transcription factors of signal transducers and activators of transcription (STAT) family. Suppressors of cytokine signaling proteins (SOCS) are critical negative regulators of GCSFR that inhibit the JAK/STAT pathway in a feedback loop. These proteins are primarily involved in the signal transduction pathways that GCSFR triggers, and stringent regulation of these proteins is integral for maintaining optimal expression of the receptor. Timely regulated initiation of the expression of GCSFR plays a central role in the sequence of events that lead to lineage divergence and in the establishment of malignancies (16). While the role played by GCSFR in neutrophil maturation and signaling is widely known, more recent work has shown considerable effects of GCSFR signaling in a wide variety of immune and non-immune cells. The results of signal transduction through GCSFR are also found to be dependent on both ligand concentration and cell location.

**Figure 2:**
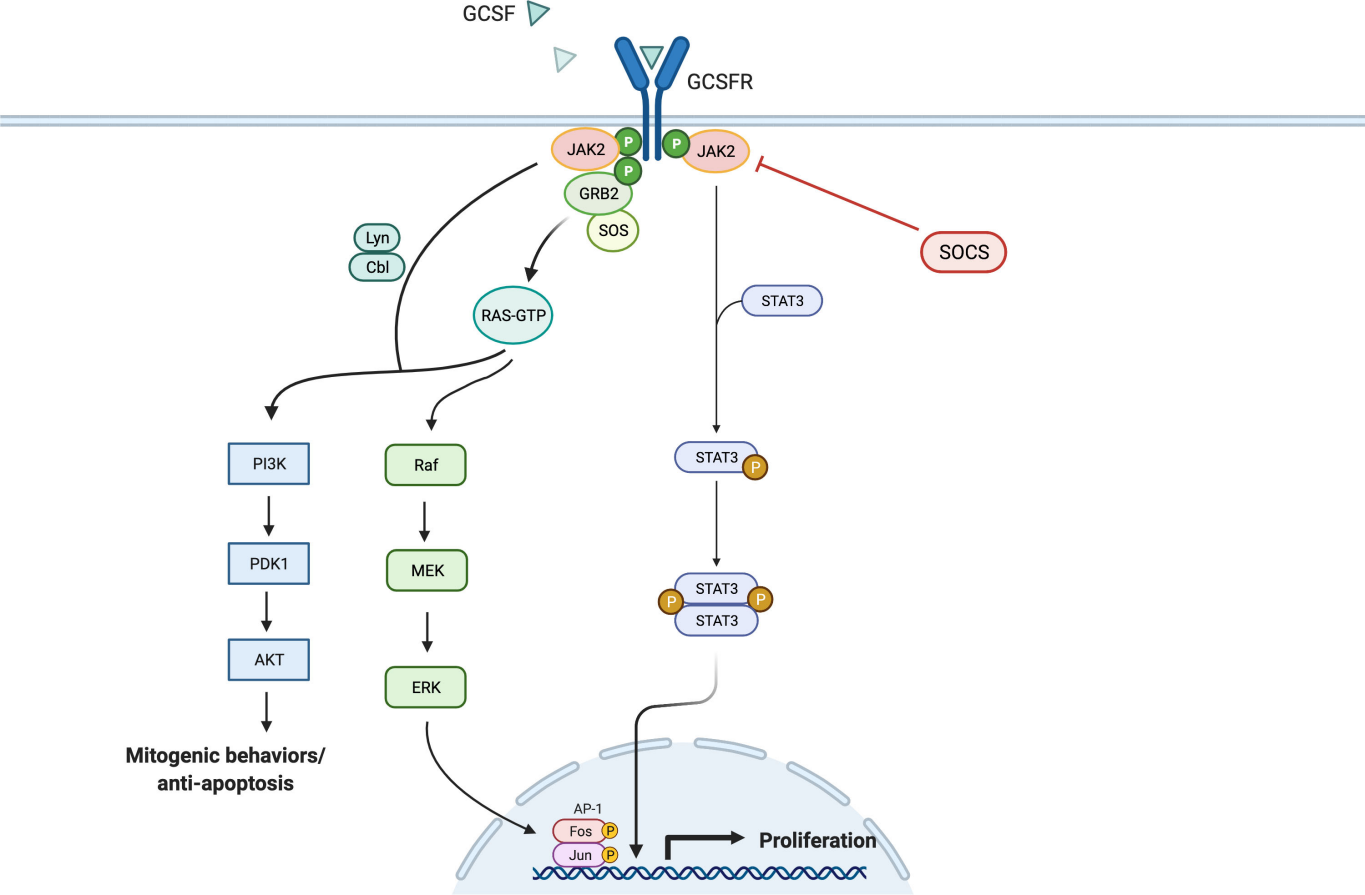
General Overview of GCSFR Signal Pathways. Adapted from “Hippo Pathway in Mammals”, by BioRender.com (2022). Retrieved from https://app.biorender.com/biorender-templates.

Like the interleukin-6 (IL-6) activation pathway, GCSF binding to GCSFR activates the signal transduction pathways by primarily inducing tyrosine phosphorylation of the receptor, which activates the JAK/STAT pathway (**Figure 2**). GCSFR has no intrinsic tyrosine kinase activity. However, upon GCSF binding, four conserved tyrosine residues in the cytoplasmic domain develop an increased affinity to STAT3, the adapter proteins Src homology and collagen homology (Shc), growth factor receptor bound protein 2, and suppressor of cytokine signaling 3 (SOCS3) after being phosphorylated by JAK1, JAK2, and Tyrosine kinase-2 Tyrosine kinase-2 (TYK2) (17). The cellular signal is propagated further when JAK2 recruits another tyrosine kinase, Lyn protein, a key inducing factor for the mitogenic behavior of GCSFR. Lyn, which directly binds to Casita B-lineage Lymphoma or Cbl, an E3 ubiquitin-protein ligase, couples Lyn to Phosphoinositide-3 kinase (PI3) (18, 19).

While JAKs are phosphorylated as a result of the GCSF ligation of GCSFR, STATs are simultaneously activated and traffic downstream signals. Like JAK proteins, STAT proteins have important tyrosine residue sites that need to be successfully phosphorylated to be activated. There is extensive evidence that the tyrosine residues in the membrane-proximal cytoplasmic region of GCSFR, Y704 and Y744, are integral for STAT activation *via* a direct docking mechanism at those sites (20). Three distinctive STAT proteins are involved in this activation step: STAT1, 3, and 5. While all three play roles in the activation of cellular proliferation, each of the three has a distinct mechanism of action. Phosphorylated STAT1 (pSTAT1) controls the dormant stem cell’s entry into the cell cycle and stimulation of interferon (IFN) for an inflammatory response. Phosphorylated STAT3 (pSTAT3) acts as a mediator and a key regulator of pluripotent cell maintenance. Phosphorylated STAT5 (pSTAT5) acts as a downstream messenger that induces the activation of the erythropoietin receptor (EPOR) in the setting of basal GCSFR activity (21, 22). STAT3 activation is required for GCSF-dependent granulocytic differentiation and regulation as the protein leads to sustained GCSF-induced proliferation in certain myeloid cell lines. This is demonstrated by the fact that reduced STAT3 signaling led to the loss of stem cell maintenance, while STAT5 and STAT1 largely affected cellular survival (23). STAT3 seems to hold greater importance in the signal transduction process, as it is expressed in greater amount than STAT5 and STAT1 in GCSF/GCSFR-dependent signaling (24, 25). Consistently, past findings differentiate STAT3 from STAT5 as the major proponent for oncogenesis in solid tumors. It has been detected in solid tumors at significantly increased levels, while STAT5 is found at higher levels in hematological malignancies. However, there is emerging evidence that shows STAT5 having a wider role in mediating solid tumorigenesis than previously thought. Although STAT5 has not been included in **Figure 2**, as it is not yet confirmed to be a potent oncogene in solid tumors, an increased expression level of STAT5 was notably found in lung cancer cells, promoting the survival of cancerous cells *via* the tyrosine-protein kinase ABL2 and transcriptional coactivator TAZ signaling axis (26, 27).

**Figure 3:**
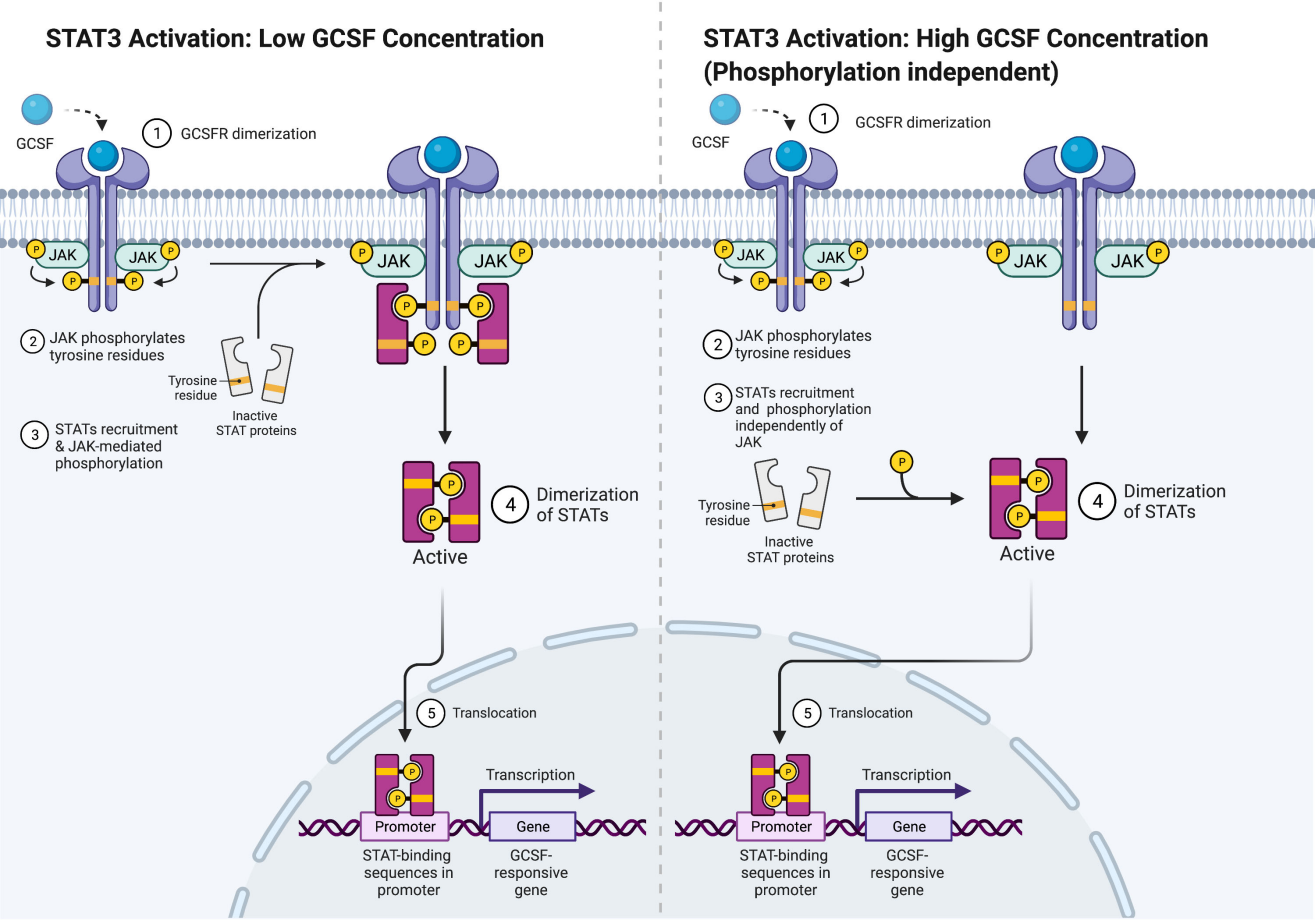
STAT3 Activation Mechanisms. Adapted from “Cytokine Signaling through the JAK-STAT Pathway”, by BioRender.com (2022). Retrieved from https://app.biorender.com/biorender-templates.

For STAT5 and STAT1, conserved Box 1 and Box 2 motifs in the cytoplasmic domain of GCSFR are required for activation (28, 29). Mutational analyses of the mouse GCSFR cytoplasmic domain elucidated the importance of conserved Box 1 and 2 sequence motifs in GCSF-mediated receptor growth signaling. These motifs are located at the carboxy-terminal end of the receptor close to the membrane-proximal 53 amino acids of the cytoplasmic domain and act as a latching site for tyrosine-specific phosphorylation of the transcriptional regulator p75^c-rel^ in Ba/F3 transformants—a process integral for GCSFR growth signal transduction (30). However, STAT3 does not rely on the conserved motifs for activation. Instead, it acts through tyrosine-dependent and tyrosine-independent mechanisms for activation depending on the ligand concentration (**Figure 3**). Recently, a comparison of STAT3 activation between wild-type (WT) GCSFR with the deletion mutants d715 and Y704F suggests STAT3 activation has an alternate mechanism of activation at low GCSF concentrations. At low concentrations, STAT3 activation is mediated by the phosphorylation of the Y704 and 744 sites by receptor-associated JAK kinase family members, leading to dimerization mediated by reciprocal Src homology 2 (SH2)-pY705 motif interactions and then nuclear translocations and binding to specific DNA elements of *CSF3R* (31). Possible explanations for these two sites acting as the major docking sites may be that they allow more efficient phosphorylation or have higher affinity than the putative intermediate docking protein (32). In contrast, GCSFR is activated independently from intracellular tyrosine at the saturating concentrations of GCSF (100 ng/ml) (15). Under these high ligand concentration conditions, STAT3 activation is mediated by a mechanism involving the C-terminal region of the full-length GCSFR, removing the need for the tyrosine docking sites. The evidence suggests that emergency granulopoiesis in response to high levels of GCSF may be accomplished through an independent signaling pathway mediated by the distal region of GCSFR without the requirement of phosphotyrosine residues.

Unlike STAT3, STAT1 and STAT5 can be activated when a ligand binds to their receptors in the absence of receptor tyrosine phosphorylation. It is currently thought that JAK1 and JAK2 recruit and phosphorylate STAT1 and STAT5, respectively, in a direct manner. The expression of STAT5 is carefully regulated by Src homology phosphatase-1 (SHP1). A comparison of SHP1^WT^ and SHP1^mut^ expression in 32D cells, which are murine pre-B cells that require the cytokine for growth and survival, led to the finding that SHP1 directly regulates the intensity of GCSF-mediated proliferation in a negative manner through direct association with Y729 and indirect interaction with phosphorylated Y729 of GCSFR carboxy terminus. Although the SH2 domain of SHP1 did not interact with phosphorylated tyrosine residues in an *in vitro* binding assay, Src homology phosphatase-2 (SHP2) still maintained its regulation on proliferation at the expense of GCSF-induced differentiation (31, 33). This could possibly be explained by the fact that STAT5 has three active isoforms: STAT5A, STAT5B, and STAT5p80. While STAT5A and STAT5B bind to the proliferation-specific domain of GCSFR, STAT5p80 binds to phosphorylated Y704 of GCSFR, which is essential for differentiation (28, 34). The activated STAT5 is now able to behave similarly as STAT3 in that it is able to cause STAT5 dimerization and attract transcription factors like lymphoid enhancer-binding factor-1 (LEF1) and C/EBPα to the nucleus (35, 36). Investigations using samples from congenital neutropenia (CN) patients with and without AML revealed that higher levels of phosphorylated STAT5 and LEF1 were found in CN patients who developed AML subsequently than in CN patients who did not develop AML. Furthermore, a recent study on breast cancer gene-1 (BRCA1) associated with ovarian cancers revealed a role for STAT5 in mediating solid tumorigenesis. Upregulated STAT5 inhibited the transcription factor p21, a cell-cycle inhibitor, leading to increased proliferation of ovarian carcinomas (37). Furthermore, studies on JAK2 V617F mutations, acquired somatic mutations often found in patients with myeloproliferative cancers, revealed that STAT5 over-activation can also cause increased cell proliferative behavior in non-myeloid cells such as mammary cells (38). A point mutation in JAK2 allowed constitutive activation of JAK2 in epithelial mammary cells, which led to hyperactivation of STAT5 that eventually enhanced the proliferation of epithelial memory cells. These findings highlight a greater role for STAT5 in the oncogenesis of both solid tumors and hematopoietic cancers.

Similar to STAT5, STAT1 activation is dependent on the successful formation of STAT1 homodimers by reciprocal phosphotyrosine–SH2 domain interactions, which allows for translocation of the homodimers to the nucleus, which is followed by binding to the promoters of the targeted genes (39). STAT1 is tightly regulated, as its response is rapid and transiently activated in response to ligand stimulation. It is also subjected to regulation by SHP1 and SHP2 in a negative regulatory manner. Both phosphatases reduce JAK/STAT1 signaling by inactivating the interferon receptors and JAKs through dephosphorylation (18, 23).

**Figure 4:**
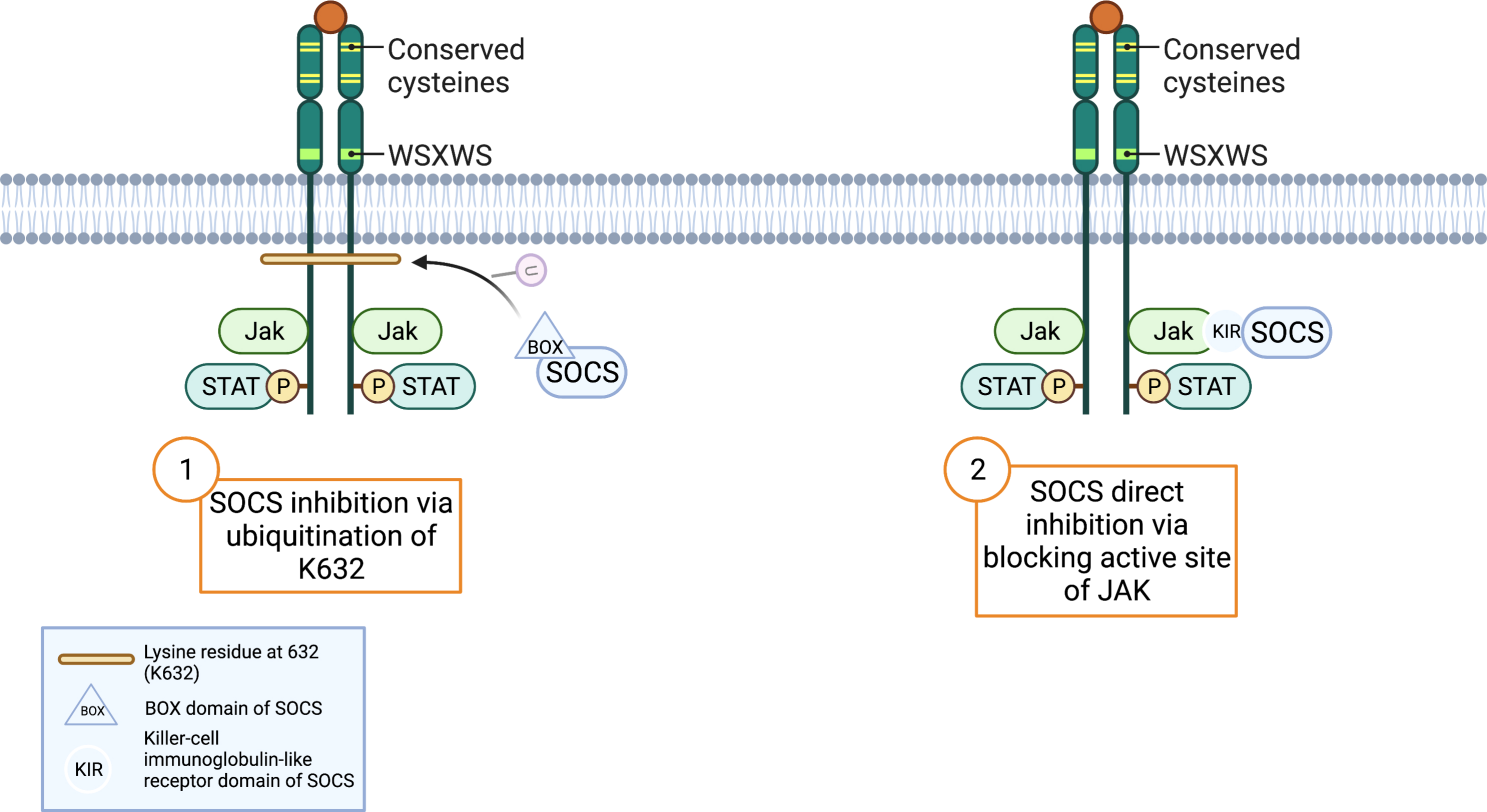
SOCS Inhibition Mechanisms. Adapted from “Cytokine Receptor Families”, by BioRender.com (2022). Retrieved from https://app.biorender.com/biorender-templates.

In earlier paragraphs, secondary GCSFR regulators specific to each protein were explored, but SOCS proteins are the primary regulators of GCSFR. While STAT signaling regulates the intensity of signal transduction induced by GCSF, members of the SOCS family control the duration of the signal. STAT activation induces the expression of SOCS, and in turn, SOCS inhibits the signaling cascade in a classic negative feedback loop. While there are eight proteins in the SOCS family, SOCS1 and SOCS3 are currently at the center of interest, as they are unique in the SOCS family for their particularly short N-terminal domain, which allows direct interaction with JAKs to inhibit the catalytic activity. SOCS1 and SOCS3 have two described mechanisms of inhibition. First, the SH2 domain of SOCS3 directly binds to the phosphorylated activation loop of JAK and the killer-cell immunoglobulin-like receptor (KIR) domain, which then blocks the active site of JAK. Second, the elongin B/C heterodimer and ternary complex-bound SOCS box domain interact with Cullin 5 (CUL5) to form the scaffold of an E3 ubiquitin ligase that ubiquitinates both JAKs and GCSFR, marking them for degradation by proteasomes. (**Figure 4**) (40).

The complex signal transductions and physiological effects driven by the GCSF/GCSFR system were recently investigated using a global GCSF knockout mouse model by Zhang et al. to determine how GCSF signaling modulates the physiological effect of non-alcoholic fatty liver disease (NAFLD). Overall, GCSF deficiency in mice alleviated a high-fat diet (HFD) and palmitic acid (PA) induced obesity, hepatic steatosis, and insulin resistance. A comparison of isolated primary hepatocytes from both GCSF knockout (−/−) and WT (+/+) mice treated with either an HFD or a standard-chow diet (SCD) revealed that administration of exogenous GCSF significantly aggravated palmitic acid-induced lipid accumulation in both the GCSF knockout (−/−) and WT (+/+) mouse samples. The model also showed a physiological difference in GCSF−/− mice having significantly lower liver weight, a lower mass of epididymal white adipose tissue, and a lesser extent of hepatic steatosis than their control littermates after 13 weeks of HFD feeding before the introduction of exogenous GCSF (41). These findings were confirmed by intrahepatic triglyceride content from hepatic and cellular triglyceride assay, hematoxylin and eosin staining, and oil red O staining from the histological analysis. With the use of serine–threonine kinase (Akt) and glycogen synthase kinase-3 (GSK3) as markers for insulin sensitivity and glucose tolerance, Western blotting showed significantly increased phosphorylation of JAK1/2, STAT3, Akt, and GSK3 in the livers of HFD-fed GCSF−/− mice exposed to exogenous GCSF treatment. Consistently, decreased SOCS3 was detected in these mice, suggesting that GCSFR may be able to regulate lipid metabolism and insulin sensitivity *via* JAK/STAT3 signaling to modulate NAFLD. Immune cells in the liver through immunohistochemical staining and flow cytometry using myeloperoxidase as a marker for neutrophils and F4/80 for macrophages were also compared between GCSF−/− mice and WT prior to GCSF treatment.. Both neutrophils and macrophages were significantly decreased in the livers of HFD-fed GCSF−/− mice compared to WT, suggesting an alternate pathway in which GCSFR can indirectly affect the development of NAFLD by regulating the production and mobilization of neutrophils in the absence of GCSF (41). This study identifies the importance of GCSFR regulation in the presence of GCSF in response to diet-induced changes in hepatocyte metabolism. The study also suggests that increased GCSFR deregulates JAK/STAT/SOCS signal pathway, which can bring immunomodulation that may attenuate the hepatic metabolism process (41, 42).

### Signal modulation

Whether GCSFR is susceptible to typical receptor translational modification remains investigated. However, emerging evidence suggests several mechanisms play functional roles in regulating the receptor. Specifically, C-mannosylation regulates the receptor by modulating its signaling. A post-translational modification that occurs intracellularly in the endoplasmic reticulum before protein folding and secretion, C-mannosylation regulates protein folding, guidance of substrate of proteins, and cellular signaling. This mode of protein modification has been found to be functional in regulating the downstream signaling of GCSFR. In the receptor, C-mannosylation at W318 regulates granulocytic differentiation in myeloid 32D cells and affects GCSF-dependent downstream signaling by changing ligand binding capability. Investigators used transduction of myeloid 32D cells with WT or W318F GCSFR expressing lentivirus to show that the absence of this C-mannosylation site on GCSFR resulted in lower phosphorylation levels of STAT3 compared to WT-expressing cells as well as a lower number of differentiated cells (43). The *in vitro* experiment also confirmed that C-mannosylation regulates the JAK/STAT pathway by affecting the capability of ligand binding without any change in the cell surface localization of GCSFR, resulting in myeloid cell differentiation (44).

## Regulation of granulocyte colony-stimulating factor receptor expression

Initially, it was thought that GCSFR was expressed only on myeloid and hematopoietic stem cells. However, GCSFR has been shown to be expressed on epithelial cells, endothelial cells, ganglion cells, neurons, cardiomyocytes, and numerous cancer cell lines (45–48). Furthermore, the expression of GCSF and GCSFR is increased in several types of solid tumors including breast cancer, bladder cancer, GI cancers, and gliomas (8, 49–51). While GCSFR regulation has primarily been studied in myeloid cells, here we will discuss what is also known about GCSFR regulation in both myeloid and non-myeloid cells. By understanding how GCSFR expression is regulated, we will have greater insight into why and how dysregulation occurs in tumor development.

### Regulators of transcription

The first barrier to transcriptional activation is chromatin accessibility. Physical access to chromatin is regulated through the topological organization of DNA binding proteins like nucleosomes and other chromatin binding factors (52). Post-translational modifications of nucleosomes contribute to chromatin accessibility, which can restrict or promote transcription factor binding. Early investigations of the methylation status of GCSFR promotors suggest that hypermethylation of the HpaII restriction site inhibits GCSFR transcription.

Examination of methylation patterns in lymphocytes that lacked GCSFR expression revealed hypermethylation of the promoter region of *GCSFR* gene, while macrophages, known to have high levels of GCSFR expression, exhibited hypomethylation of the promoter region. Lastly, granulocytes and monocytes exhibited no methylation (53). Taken together, these data suggest that GCSFR promotor methylation is a critical regulator of GCSFR expression. While histone remodelers, like SWI/SNF-related, matrix-associated, actin-dependent regulator of chromatin, subfamily D, member 2 (SMARCD2), and STAT5, have been shown to contribute to the regulation of GCSF-induced differentiation of neutrophil granulocytes, the histone remodeler(s) involved in GCSFR specific modifications and whether they are conserved among cell types remain a mystery (54, 55).

Transcription factors also play a critical role in regulating gene expression. CCAAT/enhancer-binding proteins (C/EBPs) are a family of transcription factors that increase the transcription of numerous genes involved in proliferation, differentiation, and survival by binding the promotor regions of target genes (56). Currently, there are six known distinct C/EBPs (C/EBPα, C/EBPβ, C/EBPγ, C/EBP00190, C/EBPδ, and C/EBPζ). Several of these factors are critical for granulopoiesis including C/EBPα, which is also critical for the differentiation of several cell types including hepatocytes, adipocytes, lung cells, and ovarian cells (57). C/EBPα binds a GCAAT site found in the promotor region of *CSF3R* in myeloid nuclear extracts, and mutations in the site reduce promotor activity by 60% (58). C/EBPα (−/−) mice exhibited undetectable levels of GCSFR mRNA, supporting a critical role of this transcription factor (59). While one group found undetectable levels of GCSFR mRNA in C/EBPα KO mice, another group of investigators found that cell lines established in the fetal liver of C/EBPα (−/−) mice expressed GCSFR mRNA, which increased with the addition of granulocyte-macrophage colony-stimulating factor (GM-CSF), suggesting a mechanism of GCSFR expression independent of C/EBPα (60). Rat sarcoma virus (RAS) signaling enhances the ability of C/EBPα to transactivate the GCSFR promotor by phosphorylation of S248 of the C/EBPα transactivation domain in the U937 myeloid cell line and 293T embryonic kidney cells. Furthermore, PKC blocks this activation (61). C/EBP00190 can also regulate GCSFR when transiently transfected into HeLa cells, suggesting a potential additional level of regulation (57).

**Figure 5:**
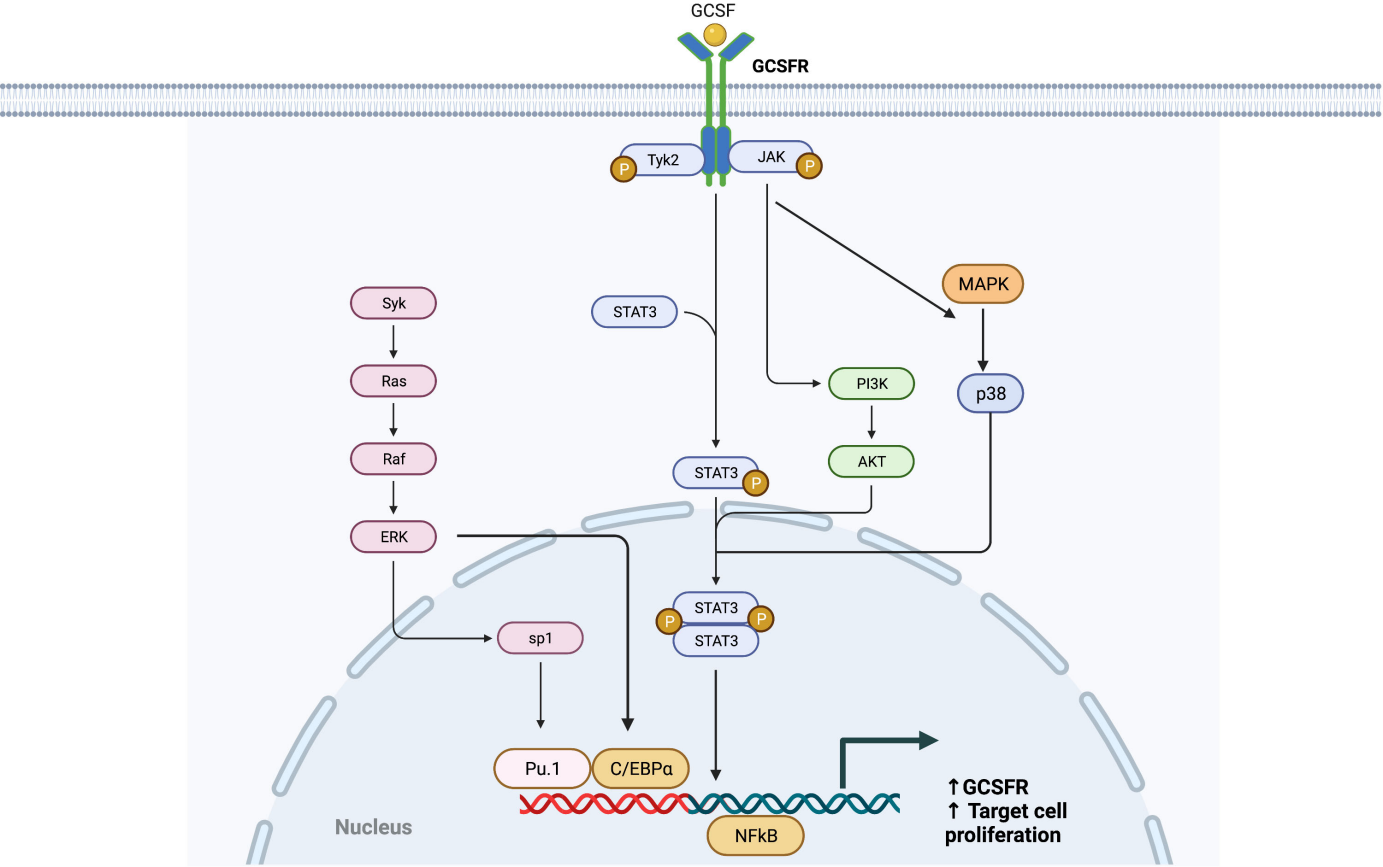
Transcription Factors of GCSFR (CSF3R). Adapted from “CREB Signaling Pathway”, by BioRender.com (2022). Retrieved from https://app.biorender.com/biorender-templates.

Two additional transcription factors have been identified that are involved in the regulation of GCSFR. PU.1, an ETS-family transcription factor encoded by the *Spi1* gene, is a key differentiation regulator that can alter the expression of thousands of genes involved in hematopoiesis including GCSFR (62, 63). PU.1 binds a purine-rich DNA sequence (5′-GAGGAA-3′) called the PU-box located at +36 and +43 in the 5′ untranslated region of the GCSFR promotor. Mutation of this region reduces promoter activity by 75%. Additionally, C/EBPα physically interacts with and activates PU.1 distal enhancer in myeloid differentiation, suggesting an additional level of transcriptional complexity (64). Interestingly, when promoter activity was monitored by luciferase assay, activity increases were observed in NB4 and HL60 leukemic cell lines but not in the non-myeloid cell lines, Jurkat or BJAB (65–67). Later studies discovered an interaction between C/EBP00190 and activating transcription factor 4 (ATF4) at CEBP binding sites of the GCSFR promoter (**Figure 5**) (68). Investigators used a luciferase reporter construct in Jurkat cells to demonstrate that homodimers of C/EBP00190 and heterodimers of C/EBP00190 activate the GCSFR promotor equally well, whereas C/EBPα transcription is inhibited upon heterodimerization with ATF4 (68). These data suggest complicated and cell type-specific regulation of GCSFR expression. Many of these studies were done during the time when GCSFR was thought to be expressed explicitly in myeloid cells with later studies performed in lymphocytes. A more recent study found an additional step of GCSFR expression regulation in a neutrophilic granule protein (NGP) neuroblastoma subpopulation of CD144^+^ cells (3). STAT3, which is activated through GCSF signaling, directly regulates GCSFR expression, suggesting a feed-forward loop. Whether these transcription factors are involved in GCSFR regulation in epithelial cells and fibroblasts within tumor microenvironments remains unknown.

### Translational regulation

MicroRNAs (miRs), short non-coding RNA molecules, bind to target mRNAs and allow translational repression and gene silencing. miRs play regulatory roles in cellular processes from proliferation to apoptosis at the translation stage. In relation to GCSFR, miRs play a critical role in combatting truncated GCSFR variants, which is important, as defective receptors have been shown to confer resistance to apoptosis and contribute to oncologic transformation. Currently, several miRs have been shown to regulate GCSFR expression, and dysregulation of expression in miRs are linked to diseases.

The miR-155 is highly expressed in hematopoietic progenitor cells and several hematological malignancies. Patients with severe congenital neutropenia (SCN), who have higher levels of class IV GCSFR, are also found to have higher levels of miR-155. Itkin et al. demonstrated that miR-155 was aberrantly upregulated in a STAT5-dependent manner for individuals with a greater level of class IV GCSFR, suggesting that upregulated miR-155 can increase the risk of *de novo* leukemia or leukemia relapse for these individuals. The pro-tumor effects mediated by miR-155 upregulation include the suppression of growth factor independent-1 transcription repressor, which is crucial for myeloid differentiation and tumor suppression, and tumor protein p53 inducible nuclear protein-1, which has anti-proliferative and pro-apoptotic activities. Additionally, miR-155 indirectly promotes the secretion of C-C chemokine ligand-2 (CCL2), a strong chemotactic factor important for regulating macrophage recruitment and polarization during inflammation (69). The miR-155-mediated CCL2 upregulation was found to upregulate GCSF-induced mobilization *via* C-X-C motif chemokine-12/C-X-C motif chemokine receptor 4 (CXCL12-CXCR4) signaling axis and STAT5 activation when class IV GCSFR was present (70). This finding highlights the pro-tumorigenic implications of miR-155-mediated GCSF and GCSFR expression and increased leukemogenicity in SCN patients.

While miRNAs can regulate GCSFR expression, signaling through the GCSF axis can also increase the expression of pro-tumor miRNAs. Recent work by Zhang et al. demonstrated that GCSF treatment on the HCT-8 colon cancer cell line resulted in a gradual increase of miR-125b expression in a time-dependent manner (71). Previous studies suggest that miR-125b can act in a pro-metastasis manner by modulating the tumor microenvironment *via* promotion of apoptosis and epithelial to mesenchymal transition (72). A recent analysis of colorectal cancer (CRC) patient samples with or without node metastasis confirmed that samples from patients with metastasis had higher expression of miR-125b. Further work on the HCT-8 cell line by ectopically expressing miR-125b in the cell line revealed that ectopic miR-125b could significantly promote migration and invasion of CRC cells, indicated by the transwell migration array and Matrigel invasion array. The finding was consistent with tumors of mice injected with CRC cells with overexpressed miR-125b metastasizing in the liver and lung (71, 73). The migration speed of HCT-8 cells also increased in a dependently of in miR-125b overexpression, as the wound healing assay showed much faster wound healing than that of the control. Zhang et al. performed a dual-luciferase activity assay and identified myeloid cell leukemia-1 (MCL1), an inhibitor of apoptosis that contains 3′-UTR putative target sequences for miR-125b, to be the direct target of this translational modification. The result revealed miR-125b inhibiting the relative luciferase activity of WT MCL1 3′-UTR constructs with firefly luciferase vector when co-transfected with miR-125b mimics, suggesting that miR-125b directly binds to 3′-UTR of MCL1 to inhibit its expression (71). While the exact mechanism by which miR-125b acts in CRC initiation and progression is unclear, different studies have identified the increased presence of miR-125 in breast and liver cancers, suggesting that miR-125-induced inhibition of MCL1 protein may selectively promote apoptosis-resistant cancer cells, which then can have greater metastatic potential than the cancer cells susceptible to apoptosis, in various organs (73).

## Trafficking and post-translational modifications

### Localization

Protein localization requires the accumulation of a protein at a destined site to produce cellular signaling and is an important step for signal trafficking. Endocytosis is one method by which signal transductions are modulated in terms of intensity and duration. In some disease processes, receptor trafficking can become altered, resulting in hampered receptor signaling pathways. Enhanced green fluorescent protein (EGFP)-tagged WT GCSFR demonstrated ligand-induced spontaneous receptor internalization with predominant localization in the Golgi apparatus, late endosomes, and lysosomes (74). Positions at 749–755 and 756–769 in the C-terminal region of GCSFR aid in the internalization of the receptor *via* their dileucine internalization motif, which is dependent on phosphorylation of a serine residue at position −4 to −5 upstream of the dileucine pair. The phosphorylation of this serine residue facilitates the interaction with activator protein-2 (AP2) (75). Internalization of the receptor has a synergizing effect on JAK activation. Additionally, the integrity of a crucial tryptophan residue (W650) in the juxta-membrane region of the receptor for JAK activation is found to further stabilize the internalization process.

### Recycling

**Figure 6:**
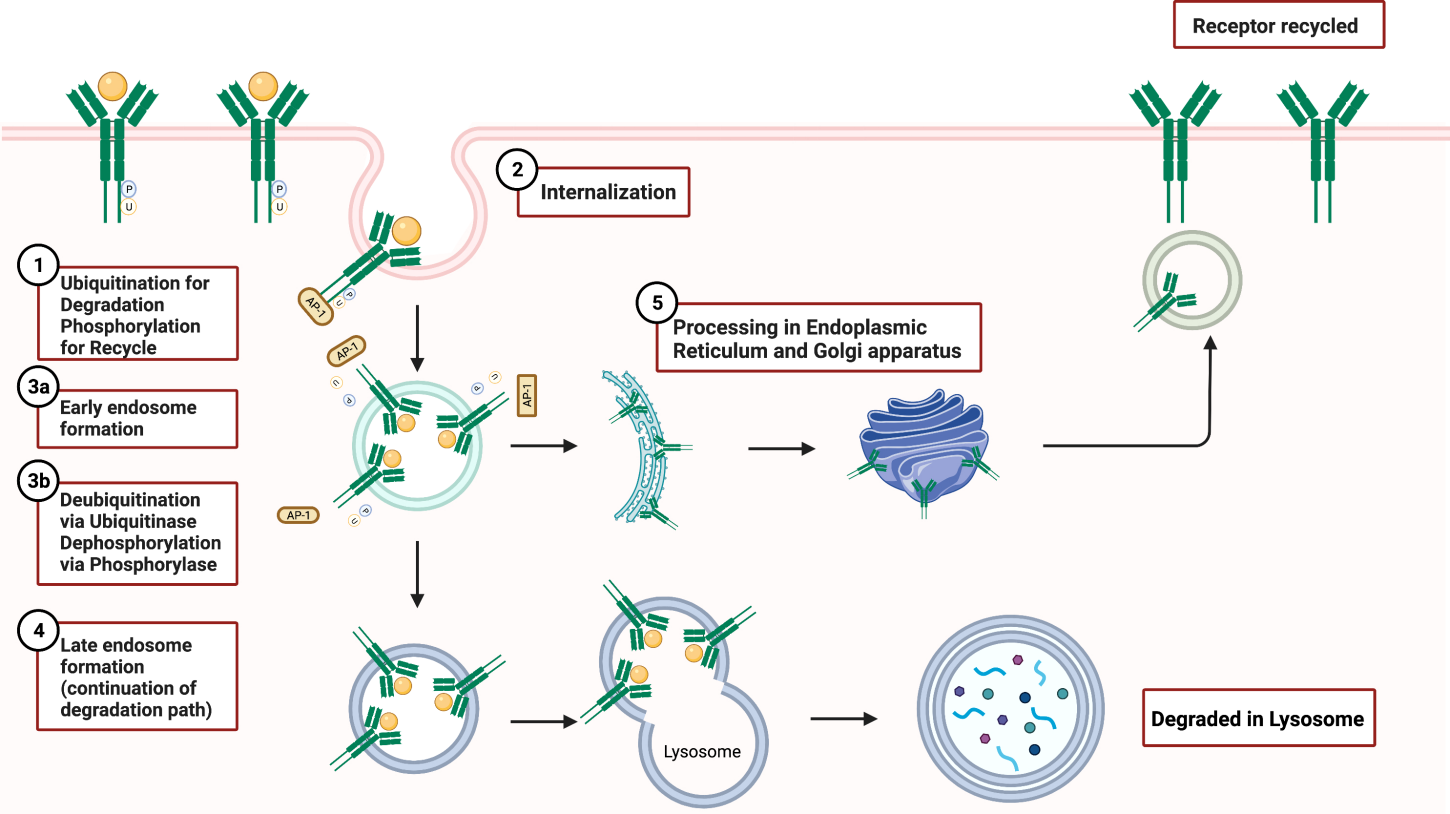
GCSFR Degradation and Recycle Mechanisms. Adapted from “Endocytosis and Exocytosis with Membrane Rupture (Layout)”, by BioRender.com (2022). Retrieved from https://app.biorender.com/biorender-templates.

The fate of internalized receptors includes receptor degradation and recycling. Receptor recycling plays an important regulatory role in signal activation and overall signal trafficking. In WT GCSFR, GCSF binding of the receptor results in receptor internalization followed by endosome formation as presented in **Figure 6**. During this process, downstream signaling continues. The receptor inside the endosome now faces either degradation or recycling. If recycled, the early endosome undertakes a dynamic system for sorting and re-exporting membrane components *via* the endoplasmic reticulum and Golgi apparatus, respectively. Understanding the mechanism of recycling helps in determining the composition of the plasma membrane and the mechanisms of normal cellular homeostasis.

Damaged endosomal recycling is often linked to a variety of diseases, including cancer and neutropenia. Vacuolar protein sorting 45 homolog (VPS45) deficiency is often found in patients with serious infections and diseases including CN, bone marrow fibrosis, and extramedullary renal hematopoiesis. VPS45, a member of the secretory/mammalian uncoordinated 18 (SM) family, is a critical regulator that orchestrates trafficking through the endosomal system and promotes the recycling of cell surface receptors. Loss of VPS45 results in the trapping of GCSFR in endosomes and impaired lysosomal delivery (76). Linked to hypo-responsiveness to GCSF due to impaired trafficking of GCSFR, the absence of VPS45 reduced trafficking of colocalized GCSFR with lysosome-associated membrane glycoprotein-2 (LAMP2)-positive late endosomes, showing a sustained accumulation of receptor in early endosomes (77). The accumulation indicated that the absence of VPS45 arrests early endosomal activity in sorting receptors for recycling or degradation. Interestingly, past research draws a closer relationship between T224A mutation in *VPS45* gene that abolishes its gene expression in SCN patients who are often susceptible to dysregulated GCSFR (76).

In GCSFR, phosphorylation of the immediate upstream serine residue at 749 of carboxyl terminus (S749), positioned four residues downstream of the dileucine motif, is found to be a crucial determinant in the switch from slow constitutive endocytosis to fast, ligand-induced endocytosis (74). A mutation of the leucine in internalization motif-1 to alanine (L753754A) has been shown to elicit a significant reduction in GCSFR internalization, suggesting that the upstream leucine residue plays an integral role in both localization and internalization of the receptor (43). The internalization rate of WT GCSFR was compared to receptor mutants S749A and S749D that mimic an unphosphorylated lysine residue and a phosphorylated residue, respectively. Both WT and S749D GCSFR had internalized approximately 60% of the surface GCSFR within 5 min of incubation at 37°C, while only 20% of the cells expressing S749A were internalized at that timepoint. Both S749A S749D GCSFR mutant cells were not affected by spontaneous internalization of anti-GCSFR antibodies when compared with WT GCSFR cells, indicating that the phosphorylation of S749 is important in determining the rate of ligand-mediated GCSFR internalization but is not sufficient by itself to catalyze the internalization rate in the absence of GCSF (77, 78).

### Degradation

The degradation mechanisms of GCSFR are similar to most cell surface protein processing and include glycosylation and targeted ubiquitination. Derangements in the degradation process of GCSFR are found in patients with SCN and AML, which increase in GCSFR induces hypersensitivity and enhanced growth response to GCSF. (79–81).

Degradation of GCSFR begins with ubiquitination modulated by *O*-glycosylation. The cluster of threonine residues proximal to amino acid position 618 is an important site for glycosylation. The glycosylated wild-type GCSFR is expressed at the cell surface and triggers ligand-dependent tyrosine phosphorylation (82). The phosphorylation then leads to ubiquitination for proteasomal degradation. At this step, JAK2 levels decrease to limit the signaling (83). Additionally, *O*-linked glycosylation decreases the dimerization of the receptor due to its bulky charged group, which sterically hinders the process. A recent finding elucidates a novel avenue of aberrant signaling of GCSFR when the degradation signal is compromised. Threonine residue at the 618 (T618) site of the proximal membrane region of the receptor, a part of the *O*-linked glycosylation cluster, is an important motif for endocytosis and degradation. Truncation in T618 directly prevents *O-*glycosylation of the receptor and increases receptor dimerization, highlighting the receptor’s ability to be activated in a ligand-independent manner when T618 is compromised (84). Point mutation analysis of T618I mutant confirmed that this mutation prevented *O*-glycosylation of the receptor (82). Cells expressing the membrane-proximal *CSF3R* T618I mutation exhibited high rates of growth in the absence or presence of ligand without any change over the concentration gradient (85). This finding was consistent with previous reports that showed the T618I mutation causing rodent bone marrow colony formation in the absence of GCSF (86). The ligand-independent nature of T618 mutant underscores the relative potency of the truncation mutation and further highlights the importance of the threonine cluster in the function and regulation of GCSFR signaling.

The receptor is also susceptible to degradation through SOCS3-driven lysosomal degradation, in which ubiquitination of specific lysine residues in the conserved juxta-membrane motif plays a large role in regulating degradation. Unlike glycosylation, which partly inhibits JAK kinase activity, ubiquitination of the lysine residue at position 632 of juxta-membrane (K632) drives lysosomal degradation and targets STAT5 by downregulating and attenuating phosphorylation activity (**Figure 4**) (87). Covalent bonding of ubiquitin to a cytoplasmic lysine residue in GCSFR attracts lysosomal sorting effectors and proteins such as the hepatocyte growth factor regulated tyrosine kinase substrate, endocytic adaptor proteins (epsins), and the endosomal sorting complex required for transport machinery (ESCRT) complexes to create a binding site for membrane phosphoinositides. Subsequently, EAP45/Vps36 interacts with this complex to sort cargo proteins to the luminal vesicles of endosomes. SOCS’s innate ability to inhibit phosphorylation strengthens the effect of this downregulation pathway on GCSFR (39). Additionally, the lysine residue in the receptor holds importance in regulating the GCSFR-stimulated signal transduction. A lysine lacking GCSFR mutation is strongly associated with prolonged receptor expression, leading to unregulated cellular proliferation. Comparing the STAT phosphorylation activity between WT whole cell lysates and K762R/GCSFR transfectants, immunoblot analysis showed rapid diminishment of phosphorylation of both STAT3 and STAT5 in the WT compared to the mutated GCSFR counterpart 2 h post-GCSF stimulation. Akt signaling pathway, an important pathway for cell survival and proliferation, was also found to have prolonged activation in K762R mutants as compared to WT GCSFR transfectants in which Akt activity was undetected at 60 min (79).

Other biological inhibitors for degradation have been identified including methyl-β-cyclodextrin, hyperosmotic sucrose, severely reduced internalization-defective GCSFR mutants like D715, and GCSFR deletion mutations, which are often found in patients with neutropenia. Degradation inhibitors like MG132 and Bafilomycin-A take a more direct approach to restore GCSFR protein levels by preventing degradation. MG132, an effective reversible proteasome inhibitor, can readily permeate through the cell membrane and selectively inhibit proteosome machinery by attaching its peptide aldehydes to the lysosomal cysteine domain of proteases. Bafilomycin-A, a macrolide antibiotic, inhibits GCSFR degradation through acidification of either the extra cellular environment or intracellular organelles, denaturing lysosomes by specifically targeting vacuolar-type hydrogen ATPase (V-ATPase) (88).

## Protein interactions

The GCSFR-driven signal transduction mechanism is complex. Its ability to contribute to proliferation and cellular differentiation signaling in different organs is a testament to the versatility of the receptor and highlights the potential for deleterious effects when GCSFR is upregulated. The *in vitro* investigation of GCSFR in hepatocytes discussed in the *Signal Transduction* section of this review reflects the organ specificity of GCSFR-stimulated signal transduction and highlights the vast presence of GCSFR in the human body. In the liver, GCSFR regulates hepatic lipid metabolism through downstream signaling activation of the JAK/STAT/SOCS pathway. GCSFR activation induced expression of SOCS3, which then inhibited JAK activation and limited STAT3 phosphorylation, negatively regulating GCSF response (41). This negative feedback pathway had a direct influence on instigating hepatic steatosis by inhibiting the expression of Akt and GSK3, which evoke insulin insensitivity, highlighting the intracellular interplay between organ-specific proteins and the GCSFR-mediated signaling proteins (89).

GCSFR is known to interact with transmembrane proteins involved in signal transduction pathways of cells to maintain healthy homeostasis. One of these interactions is with integrin α9β1. This transmembrane protein is often found in the epithelium and aids in the translation of extracellular signals that change cell behavior, specifically cell adhesion and migration (90). α9β1 is also prominently expressed on human neutrophils and mediates neutrophil migration through vascular cell adhesion molecule-1 (VCAM1) and tenascin-C (TNC). α9β1 improves the responsiveness of GCSFR to GCSF and promotes stimulation of the cascading signaling pathway by directly interacting with GCSFR. Comparing ltgα9 WT and ltgα9−/− bone marrow cells revealed that the STAT3 phosphorylation resulting from GCSF stimulation was significantly reduced in α9-deficient cells (91). While the specific mechanism remains unclear, the permissive role of α9β1 in the GCSFR-signaling pathway as indicated in the study suggests α9β1 is important for granulopoiesis, especially in enhancing the activation of STAT3.

Another important protein interaction of the receptor is with E6-associated protein (E6AP), a ligase protein best known for ubiquitinating the transcription factor p53. E6AP targets GCSFR for ubiquitin-mediated proteasome degradation, attenuating the receptor’s function (88). GCSFR and E6AP are co-localized together in the cells, and the co-localization is enhanced in the presence of the proteasome inhibitor MG132 both *in vitro* and *in vivo* (88). E6AP is also found to promote early degradation of GCSFR, reducing the GCSFR signaling indicated by reduced STAT3 phosphorylation. Investigators determined the half-life of GCSFR in the presence and absence of E6AP by inhibition of *de novo* protein synthesis with cycloheximide. E6AP markedly reduced the half-life of GCSFR, while the half-life of T718 GCSFR mutant was modestly affected, highlighting the importance of the protein–protein interaction between E6AP and GCSFR (88, 92). The study further implicates the possibility of E6AP as an effective GCSFR inhibitor to treat GCSFR upregulated diseases.

## Receptor expression and response to granulocyte colony-stimulating factor in non-myeloid cells

Previously established understanding of GCSFR implicates that the receptor can interact with non-immune cells in different organs. In recent years, the receptor and its substrate have been detected on the surface of other microvascular murine endothelial cells originating from the thymus, brain, heart, and skin, as well as other non-hematopoietic cells (3, 5, 78). In the endothelial cells of different organs, GCSFR is expressed at similar levels as in myeloid cells and acts similarly in aiding the cellular proliferation and migration of cells (93). The signaling mechanism of rh-GCSF and the receptor (rh-GCSFR) in the ovarian adenocarcinoma cell line, HEY, allows a better understanding of GCSFR functioning beyond its typical role.

The *in vitro* model of rh-GCSFR in HEY constructed by Brandsetter et al. showed the active participation of the receptor in mediating mitogen-activated pathway (MAP). To demonstrate this, proliferative and differential signals were induced *via* GCSFR, and a similar signal transduction mechanism was shown in the model. Y646, 744, and 764 sites were important for activating JAK kinases and activating p21^Ras^/MAP kinases. Upon exogenous GCSF stimulation of HEY cell lines, *AP-1-(c-Jun/c-Fos)* regulated gene accumulated and upregulated *CSF3R* expression by 40% (94). Three MAP kinase groups were involved in MAP: p38 kinases, the extracellularly regulated kinases (ERKs), and the c-Jun N-terminal kinases (JNKs) found in stress-activated pathways. Two methods by which these proteins are activated were identified: stress-associated and non-stress-associated pathways. Although these two pathways result in increased proliferation, the stress-associated pathway involves the JNKs and p38 kinases that are activated in response to inflammation. The non-stress-associated pathway involves cytokine activation of ERKs, which can phosphorylate c-jun, an integral component of AP-1 complexes that regulate transcriptional activity (95).

In addition to its proliferative role, the retinal ganglion cell (RGC) axotomy model used by Frank et al., highlights the receptor’s neuroprotective nature in RGCs. Its constitutive expression in RGCs aids in the survival, differentiation, and proliferation of neutrophilic lineage cells. The investigators demonstrated that GCSF-mediated GCSFR expression protected RGCs from degeneration after transection of the optic nerve in a rat model (47). GCSFR-driven activation of RAS/RAF/ERK or PI3K/Akt kinases is understood to inhibit apoptosis through inhibition of caspase and by activating neurotrophins, potentially explaining this protective effect (96).

Regeneration of skeletal and cardiac muscle cells links proliferation of cells to cellular inflammatory response mediated by GCSFR upregulation. Examining rodent embryos using immunostaining, GCSFR expression was demonstrated to be increased at the period when early skeletal myocytes began differentiating and the expression of the receptor was affected by the autocrine GCSF signaling as myoblasts developed (97). A serial histological analysis up to 28 days after injury (inducing stress by injecting cardiotoxin directly into rodent femoral muscle cells) demonstrated the synchronous nature of inflammatory response and upregulation of GCSFR to protect cells and prevent apoptosis. GCSFR expression was observed *via* immunofluorescence on day 5 after cardiotoxin injection. Day 5 corresponded to the same day the skeletal muscle progenitor cells or satellite cells (SCs) began proliferating, confirming that increased expression of GCSFR coincides with the proliferation period of cells (97). Furthermore, in the isolated myofiber samples of day 5, 94.4% of activated SCs or migrating SCs into the injured site with syndecan-4 (SDC4) activation showed increased expression of GCSFR. This suggests that both activated SCs and inflammatory factors were present at the same time GCSFR expression increased during the first cellular proliferation (98). Upon the activation of GCSFR, the same important signal trafficking proteins seen in myeloid cells like ERK, JNK, p38MAPK, Akt, and STATs were activated. The level of expression of these proteins paralleled with the upregulation level of GCSFR, promoting the proliferation of myoblasts (97). A similar expression pattern of GCSFR was shown in post-myocardial infarction (MI) cardiomyocytes. Additionally, upregulation of the receptor in myocardial infarction cardiomyocytes and cardiac fibroblasts of cultured rodent cardiomyocytes evoked similar protective and anti-apoptotic roles for the damaged cells *via* the JAK/STAT pathway by producing angiogenic factors (99).

While these findings highlight the cell-protective characteristics of GCSFR signaling in cells, they also implicate the damning concern that GCSFR signaling can inhibit the cellular apoptosis mechanism to encourage cancer cells to grow (96).

## Cancer

It is evident that unbridled expression of GCSFR causes unnecessary and possibly dangerous cellular proliferation and differentiation through complex downstream signal transduction. Its ability to induce proliferation pathways led investigators to look closely at GCSFR functioning in human tumor cells to better understand the relationship between upregulated GCSFR and different cancers. Wojtukiewicz et al. reported the detection of high GCSFR expression in 20 out of 28 assessed breast cancer tissue samples. Immunoblotting showed high GCSFR expression on the endothelial cells (ECs) of small blood vessels supplying breast cancers in those 20 samples, suggesting the possibility of GCSFR aiding in the proliferation and migration of ECs by supporting angiogenesis in breast cancers. Furthermore, co-expression of GCSFR with vascular endothelial growth factor (VEGF), VEGF receptor, and tissue factors were found in those samples, highlighting the interplay between the receptors for angiogenesis promotion and in providing mitogenic support for the progression of malignant cells. Similar to breast cancer, higher expression of GCSFR is found in nasopharyngeal, oral cavity, colorectal, gastric, and ovarian cancers, solidifying the relationship between increased levels of GCSFR expression and solid tumors (5, 8, 10, 84).

Many investigators have demonstrated a link between the overexpression of GCSFR and pro-tumor effects in numerous cancers such as neuroblastoma and CRC (3, 51, 99). In the central nervous system, endogenously expressed GCSFR was found to be upregulated in response to external stress-related stimulation such as nerve injuries or hypoxia, a common feature of the tumor microenvironment (100). An *in vitro* mechanical scratch neuronal injury model showed upregulation of GCSFR in spinal cord capillaries compared to the control sample. The model also highlighted the functioning of nucleophosmin-1 (NPM1), a neuron-specific protein, that was increased alongside GCSFR to reduce apoptosis by inactivating caspase-activated DNase (48). Xenograft and allograft murine models of neuroblastoma showed increased GCSFR expression promoting the proliferation and metastasis of neuroblastoma through GCSF-dependent phosphorylation of STAT3 signaling in CD114^+^ cells. Agarwel et al. reported a positive feedback loop between GCSFR and STAT3-mediated transcription of *CSF3R* (3).

Upregulation of GCSFR is also detected in glioma. While GCSF and GCSFR expressions were not detected in the normal brain cortex or primary cultured astrocytes, they were widely expressed in glioma samples (101). This finding suggests that GCSFR expression may facilitate both autocrine and paracrine modes of stimulation and maintenance of glioma. Using a bromodeoxyuridine incorporation assay, the investigators demonstrated a significant increase in proliferating glioma cells with exogenous GCSF treatment. Specifically, bromodeoxyuridine (BrdU)-positive cells were increased by 50% in GCSF-treated groups compared to groups without GCSF treatment (78). Treatment of primary cell cultures derived from glioblastoma patients and the glioma cell lines T98G, U251, and U87 with GCSFR antibody resulted in a significant decrease in the frequency of BrdU-positive cells and colony formation rate by an average of 15% compared to those without treatment (78).

The same behavior can be seen in bladder carcinomas in which tumor cells’ continuous expression of GCSF and GCSFR allowed for a functional autocrine/paracrine signaling loop that promotes the survival and growth of bladder cancer cells. This upregulation bolstered poorly differentiated proliferation as observed in multiple epithelial cancers and was a significant defining factor of the invasiveness of cancer. Higher GCSFR expression was also associated with the presence of lymph node metastasis in gastric cancers. In cultured gastric cancer cells (SGC7901), GCSFR increased proliferating cell nuclear antigen levels and induced cell proliferation. Wound healing assays have confirmed that GCSFR also increases migration in gastric cancer (49). Transfection of TCC-SUP bladder cancer cells that innately lack expression of GCSF and the receptor with full-length GCSFR resulted in a twofold increase in the proliferation rate with a sustained increase of cell survival through abrogation of apoptosis in a GCSF dose-dependent manner (102). Both the GCSFR transfected TCC-SUP and 5637-GR bladder cancer cells had increased *survivin*, a STAT-regulated gene known to mediate pro-survival functions in cells.

GCSFR is also seen cross-interacting with components within tumor stromal cells to promote tumor migration. In breast and pancreatic cancer cells, cancer-associated fibroblasts (CAFs) are most frequently found in tumor stroma not only promoting tumor progression but also inducing therapeutic resistance (103). CXCL12 signaling, known to upregulate GCSF-induced mobilization, also induces activation of CAFs, resulting in increased breast cancer stem cells (104, 105). While the specific interplay between GCSFR and CAFs remains elusive, this finding suggests a close interaction between the two. Day et al. observed GCSFR interacting with mesenchymal-lineage stromal cells in the bone marrow, CXCL12-abundant reticular cells (CAR), and osteoblasts, decreasing their capacity to support B lymphopoiesis. GCSFR was also associated with CAR expansion and support of osteogenic lineage commitment (106). However, GCSFR suppressed the production of multiple B-cell trophic factors by CAR osteoblasts, along with other cytokine factors like interleukin-6, a pro-inflammatory cytokine, and interleukin-7, hematopoietic growth factor (106).

In addition to increased proliferation of tumor cells, recent findings suggest that GCSFR promotes a microenvironment favorable for solid tumor cells to metastasize *via* immunomodulation (107, 108). Karagiannidis et al. subcutaneously injected GCSFR−/− mice with the murine colon cancer cell line, MC38, to investigate the role of GCSF signaling. They found that the GCSFR−/− mice had slower tumor growth and hypothesized that this may be due to a lack of GCSF signaling in the immune cells. The authors also noted a decrease in T cell-associated cytokine production in these GCSFR−/− mice. Real-time quantitative reverse transcription polymerase chain reaction (qRT-PCR) with RNA extracted from CD4^+^ T cells isolated from spleens of GCSFR−/− mice showed an increase in interferon-gamma (IFN*y*), which is associated with antitumor activity, and in interleukin-17 (IL-17A), which can be indicative of T-cell activation. These mice also showed a decrease in interleukin-10 (IL-10) at the mRNA level, which is indicative of regulatory T cells associated with poor anti-tumor response (108). The expression of IFN*y* and IL-17A, generally considered markers of cytotoxic CD8^+^ responses and of Th17 helper cells, respectively, were found favorable as compared to the WT, while IL-10 level decreased in the absence of GCSFR. Conversely, GCSF treatment on WT CD4^+^ cells significantly decreased IL-17A production but increased IL-10 production. These findings suggest that GCSFR directly affects T-cell phenotype and cytokine production in a GCSF-dependent manner. In *in vitro* studies, GCSFR−/− spleen-derived CD4^+^ T cells had decreased levels of the gene forkhead box P3 (*FoxP3)*, the transcription factor for T-regulatory cells. This was consistent with an increase of IL-10 and expression level of *FoxP3* in WT mice subjected to MC38 tumor injection, as compared to IL-10 and *FoxP3* levels in the GCSFR−/− mice (108). Multiplex cytokine analysis of conditioned media from cultures confirmed that IL-10 production was increased in WT mice as compared to cultures using tumor tissues from GCSFR−/− mice. This is consistent with previous studies that reported an IL-10 serum level increase in human patients with progressing CRC (73, 108). These data suggest complicated regulation of GCSFR on the immune system. The pro-tumorigenic role of GCSFR in inhibiting CD4 and CD8 T-cell responses by promoting IL-10 is recognized to play an important role in shaping the tumor microenvironment.

## Conclusion

Although GCSF has been widely used in clinics to successfully treat and prevent febrile neutropenia, a complete understanding of the complex results of GCSF signaling remains lacking. This review summarizes the available data regarding GCSFR structure, signaling, and regulation with emphasis on the role played by the receptor in diseases such as cancer. An emerging body of evidence reveals an adverse role played by GCSFR signaling in various cancers. Available evidence also shows that GCSFR activates the JAK/STAT pathway to drive the proliferation of both myeloid and non-myeloid cells. Because of this and the fact that recombinant GCSF is administered to patients with malignancy, there is an urgent need to increase our understanding of the multiple roles played by this pleiotropic cytokine beyond the well-known effects on neutrophil mobilization.

## Author contributions

All authors listed have made a substantial, direct, and intellectual contribution to the work, and approved it for publication.

## Funding

Research reported in this publication was supported by the National Institutes of General Medical Sciences of the National Institutes of Health under Award Number P20GM103639. The content is solely the responsibility of the authors and does not necessarily represent the official views of the National Institutes of Health.

## Conflict of interest

The authors declare that the research was conducted in the absence of any commercial or financial relationships that could be construed as a potential conflict of interest.

## Publisher’s note

All claims expressed in this article are solely those of the authors and do not necessarily represent those of their affiliated organizations, or those of the publisher, the editors and the reviewers. Any product that may be evaluated in this article, or claim that may be made by its manufacturer, is not guaranteed or endorsed by the publisher.

## Author disclaimer

The content is solely the responsibility of the authors and does not necessarily represent the official views of the National Institutes of Health.
